# Estimating statistical power for structural equation models in developmental cognitive science: A tutorial in R: Power simulation for SEMs

**DOI:** 10.3758/s13428-024-02396-2

**Published:** 2024-05-28

**Authors:** Elisa S. Buchberger, Chi T. Ngo, Aaron Peikert, Andreas M. Brandmaier, Markus Werkle-Bergner

**Affiliations:** 1 Center for Lifespan Psychology, Max Planck Institute for Human Development, Lentzeallee 94, 14195 Berlin, Germany; 2 Max Planck UCL Centre for Computational Psychiatry and Ageing Research, Berlin, Germany; 3 Department of Imaging Neuroscience, University College London, London, UK; 4 Department of Psychology, MSB Medical School Berlin, Berlin, Germany

**Keywords:** Structural equation modeling, Statistical power, Monte Carlo simulation, Sample size planning

## Abstract

Determining the compositional structure and dimensionality of psychological constructs lies at the heart of many research questions in developmental science. Structural equation modeling (SEM) provides a versatile framework for formalizing and estimating the relationships among multiple latent constructs. While the flexibility of SEM can accommodate many complex assumptions on the underlying structure of psychological constructs, it makes a priori estimation of statistical power and required sample size challenging. This difficulty is magnified when comparing non-nested SEMs, which prevents the use of traditional likelihood-ratio tests. Sample size estimates for SEM model fit comparisons typically rely on generic rules of thumb. Such heuristics can be misleading because statistical power in SEM depends on a variety of model properties. Here, we demonstrate a Monte Carlo simulation approach for estimating a priori statistical power for model selection when comparing non-nested models in an SEM framework. We provide a step-by-step guide to this approach based on an example from our memory development research in children.

## Introduction

Over the past decades, many psychological constructs that had originally been conceptualized as unitary entities have been shown to be multifactorial phenomena. A prominent example are the early debates about component factors of intelligence (Cattell, 1971; Horn, 1970, 1978) or personality (McCrae & Costa, 1985). However, also more specialized cognitive abilities such as executive functions (Hedden & Yoon, 2006; Miyake et al., 2000), attention (Mirsky, Anthony, Duncan, Ahearn, & Kellam, 1991) or memory (McClelland, McNaughton, & O’Reilly, 1995; Norman & O’Reilly, 2003; Schapiro, Turk-Browne, Botvinick, & Norman, 2017) are increasingly considered multi-process functions.

Understanding the underlying compositional structure of such multifaceted psychological constructs is challenging. The task becomes even more demanding when the development of component structures over time or the identification of (time-varying) differences between groups is the target of scientific investigations (Baltes, Reese, & Nesselroade, 1988). Structural equation modeling (SEM, Bollen, 1989; Jöreskog & Goldberger, 1975) provides a flexible framework that can accommodate a number of assumptions on the structure of key factors underlying a given construct and their inter-dependence. Indeed, SEM has become an increasingly popular tool in psychological science over the past decades (for a review, see MacCallum & Austin, 2000).

Since Cohen’s classic study on statistical power (Cohen, 1962), numerous researchers from psychological and neurocognitive science have stressed the issue of insufficient statistical power and its consequences on the interpretation of scientific findings in the face in inadequate sample sizes (Anderson & Maxwell, 2017; Button et al., 2013; Sedlmeier & Gigerenzer, 1989; Vankov, Bowers, & Munafó, 2014). However, determining required sample size in an SEM approach is far from trivial. While the investigation of target effects in SEM can be achieved via analytical computations (Satorra & Saris, 1985), tackling more complex research questions that rely on the comparison of non-nested models poses a challenge in determining sufficient sample sizes.

In this study, we implemented a Monte Carlo-based simulation approach and provide a step-by-step guide for conducting randomization-based analyses for a priori sample size estimations for non-nested model comparison. Specifically, we exemplify this approach with a specific research question, focusing on the componential structure of memory processes in early childhood (for theoretical background see Buchberger, Brandmaier, Lindenberger, Werkle-Bergner, & Ngo, in press). Memory developmental research represents an excellent example for this methodological approach, as researchers from different fields have put forth competing ideas on the underlying structure of memory in childhood: While a rich body of empirical work from many decades has focused on the dichotomy between episodic and semantic memory (Tulving, 1972), more recent neurocomputational theories have introduced a process-focused approach to memory development. Tenets from contemporary memory models invite the hypothesis that three kinds of neurocomputations may complementarily support adaptive memory functioning, thereby assuming a tri-partite structure of memory (McClelland et al., 1995; Norman & O’Reilly, 2003; Schapiro et al., 2017). Such competing theories on the underlying structure of memory can be addressed via model comparisons in an SEM framework. In this tutorial, we address two overarching issues that affect estimates of statistical power for such comparisons: (i) the separability of the theoretical constructs and (ii) the reliability with which the constructs are being measured. We employ this example to demonstrate the procedure and the utility of such methods, with the aim of illustrating a transferable approach to other research questions in different domains and disciplines.

### SEM in developmental cognitive neuroscience

SEM uses information about the relationship between multiple measured variables to uncover the structure of unobservable constructs, rendering this methodological framework a powerful tool in the field of developmental cognitive neuroscience (e.g., Baltes et al., 1988; Kievit et al., 2018). Most notably, it enables the translation from theories to testable mathematical models that can simultaneously include numerous observable indicators and multiple latent constructs of interest (such as behavioral constructs and neural correlates). Thus, it allows simultaneous modeling of the relationships among theoretical constructs and their co-development across different developmental windows.

In an SEM framework, theoretical constructs of interest are operationalized as latent variables that capture the underlying commonalities across a set of (measured) manifest variables. Manifest variables can directly be observed (such as items in a questionnaire or indices derived from a behavioral task) and are commonly illustrated as squares in graphical depictions of SEM. Latent variables represent hypothetical constructs that are not directly observable (such as a latent factor of intelligence or memory), but are inferred from a selection of manifest variables. In graphical depictions of SEM, latent variables are commonly shown as circles. The strength of a loading, that is, a path from a latent variable to an observed variable, represents the extent to which the observed variance is accounted for by the latent factor. In other words, the loading indicates how well a given manifest variable captures the latent construct.

The relationship among manifest and latent variables can be described in a mathematical model, which specifies all assumed parameters. Two models M1 and M2 are referred to as nested, if the parameter space in a more restrictive model M2 represents a subspace of the parameter space of the more general model M1 (Bentler & Bonett, 1980). This means that the two models only differ with regards to the specification of one or multiple parameters. Nesting of models is usually achieved via constraining free parameters from M1 to equality or to known constants. The concept of nesting represents a convenient characteristic when comparing competing models, as it allows the use of a likelihood-ratio test to evaluate their relative model fits (Satorra & Saris, 1985). When comparing non-nested models, researchers usually revert to the comparison of model fit indices that indicate how well a given model describes the data, such as the Bayesian information criterion (BIC, Raftery, 1986; Schwarz, 1978), the Akaike information criterion (AIC, Akaike, 1974), the root mean square error of approximation (RMSEA, Steiger, 2016), and the Comparative Fit Index (CFI, Bentler, 1990, but see “Dis-cussion and conclusion” for a discussion of recently proposed non-nested likelihood-ratio tests). Such indices can guide heuristic model selection, but do not allow for any statistical guarantees (e.g., a pre-specified type I error level).

### Statistical power in SEM

Over the past decades, the issue of low statistical power and its consequences for the interpretation of scientific findings has gained awareness in neurocognitive and psychological research (Cohen, 1988; MacCallum & Austin, 2000; Maxwell, 2004; Rossi, 1990). Statistical power refers to the probability of rejecting a null hypothesis, when it is indeed false (i.e., the probability of not committing a type II error) and is directly linked to the sample size of a study, the magnitude of the targeted effect, and the reliability of measurement (Brandmaier, Oertzen, Ghisletta, Lindenberger, & Hertzog, 2018; Cohen, 1988). While low statistical power decreases the scientific utility of any given study, it also decreases the likelihood that a given significant result actually reflects a true effect (Button et al., 2013). To allow well-designed empirical research to detect effects in the sample under investigation, a priori estimates of statistical power (that is computation of power estimates before conducting the study) and thus informed decisions on required sample size are crucial to prevent underpowered research (Button et al., 2013). While the desired level of statistical power can depend on specific aspects of the research question at hand, a typical convention for adequate power in the behavioral sciences is 0.8 (Cohen, 1988). However, others have argued that there is no reason to prefer type I errors over type II errors and thus one should better aim for a statistical power of 0.95, if the level of significance is kept at the conventional 5%. Note that, of course, considerations on statistical power and thus sample size estimates need to be balanced with the probability of committing type I errors, that is the probability to reject a null hypothesis if it is indeed true.

Various rules of thumb on required sample size in SEM have been recommended, including setting an absolute minimum of observations across the board (Boomsma, 1985) and adjusting to the model complexity (i.e., setting a number of observations per estimated parameter, Bentler & Chou, 1987). Unfortunately, such heuristics can be misleading, as statistical power in SEM is heavily influenced by parameters beyond the number of indicators per latent construct, e.g., the magnitude of factor loadings (Wolf, Harrington, Clark, & Miller, 2013). Further, determining the required sample size for a given study depends on the research question. One common utility of SEM is to test whether a given effect in a model exceeds a specific threshold, such as testing whether the correlation of latent factors exceeds zero, or whether a specific parameter estimate differs between groups (i.e., target effect). Another common utility is to determine how well a given model describes the data, and/or whether one model describes the data better than a competing model (i.e., model comparison). These different research questions require different types of power: the power to detect a target effect vs. the power to detect model misspecification (Wang & Rhemtulla, 2021). Further, theoretical assumptions on competing models can lead to the necessity to compare non-nested models, which prevents the use of traditional $\chi ^2$-based power estimates and therefore requires alternative approaches to determining statistical power for SEM (see “Discussion andconclusion” for a more detailed discussion of recently proposed non-nested likelihood-ratio tests).

#### Statistical power to detect a specific effect of interest

Central to many studies that employ SEM is the question of whether a specific parameter in a model (e.g., strength of a specific correlation or the slope parameter in a latent regression model) is different from a given value (e.g., Canada, Hancock, & Riggins, 2021). Imagine that a researcher aims to test whether the correlation between two latent constructs significantly differs from zero. To this end, they would compare the model fit between one model in which the correlation parameter is freely estimated and another model in which it is fixed to zero (Satorra & Saris, 1985). The corresponding null hypothesis in this case states that the parameter restrictions hold in the population. The difference in model fit will follow a $\chi ^2$-distribution with degrees-of-freedom (*df*) equal to the difference of freely estimated parameters between the two models, if the null hypothesis is true (Neale, 2000, in this example *df* = 1, because only the correlation between the two latent factors is fixed in the restricted model). If restricting the parameter of interest results in a significantly poorer model fit, this would suggest that the respective parameter indeed significantly differs from zero.

Following the logic outlined above, researchers can determine the statistical power for detecting a target effect in an SEM framework before conducting the study. A key aspect for this a priori power calculation hinges on translating the differences in a specific parameter estimate into an effect size. Such translation can be achieved by investigating the discrepancy between the model-implied variance–covariance matrices associated with (1) the population model including the “true” parameter values and (2) the hypothesized model. The discrepancy between both matrices is quantified based on a specific fit function (for details on fit functions, see Bollen, 1989). As the investigation of a target effect practically translates into the comparison of nested models, researchers can in these cases analytically determine the statistical power and use this information for decisions on required sample size (Satorra & Saris, 1985). Recently, several user-friendly tools have emerged that can guide modelers in deriving estimates for statistical power to estimate statistical power in SEM in such cases (e.g., the R packages semPower Jobst, Bader, & Moshagen, 2021; or power4SEM Jak, Jorgensen, Verdam, Oort, & Elffers, 2020 for analytical approaches; and the Shiny app pwrSEM Wang & Rhemtulla, 2021; the interactive study planner tool LIFESPAN Brandmaier, Oertzen, Ghisletta, Hertzog, & Lindenberger, 2015; or the R package simsem Pornprasertmanit, Miller, Schoemann, & Jorgensen, 2021 for simulation-based approaches).

#### Statistical power for model comparison

A second question that is of interest for many researchers – especially in developmental cognitive neuroscience – pertains to identifying the one theoretical model (from a set of competing models) that best explains the given data (Henson et al., 2016; Miller, Giesbrecht, Müller, McInerney, & Kerns, 2012; Nyberg, 1994). In the case of competing models that are nested, the analytical approach to this question (and therefore also considerations on statistical power) can be addressed analogously to the procedure outlined above (see Jobst et al., 2021 for a step-by step tutorial). However, comparing non-nested models prohibits the use of traditional $\chi ^2$-based statistics and therefore poses additional methodological challenges in determining the ‘best’ model. Nevertheless, researchers should strive for a methodological approach that matches the theoretical assumptions, rather than vice versa, that is, moving away from well-grounded theoretical considerations to meet methodological constraints. In cases where researchers aim to derive estimates of statistical power for non-nested model comparisons, randomization-based techniques offer an excellent alternative (Efron & Tibshirani, 1994). In particular, Monte Carlo simulations have proven useful to bridging this gap (Muthén & Muthén, 2002, see “How does a simulation work: Pow-er-estimations for the comparison of non-nested SEMs” for more details on Monte Carlo simulations).

### Example: Competing models on the compositional structure of memory

For many decades, the compositional nature of memory in adults as well as its maturation across development has been of great interest in psychology, cognitive science, neuroscience, and artificial intelligence research. Different models on the compositional structure of memory have been heavily debated (McClelland et al., 1995; Norman & O’Reilly, 2003; Schapiro et al., 2017; Tulving, 1972).

The most simplistic characterization of memory structure is to assume a unitary ability underlying different memory demands, akin to a g-factor of memory (Spearman, 1904). Such a unitary model of memory suggests no differentiation of component processes within declarative memory, but rather claims that the performance on various types of memory demands is grounded in a single ability factor.

An alternative prominent view is the classic dichotomy between episodic and semantic memory systems (Squire, 1987; Tulving, 1972). Such a bi-partite architecture of memory postulates a division between one component responsible for learning specific events embedded in their temporal and spatial context (episodic memory) and a second component responsible for learning regularities and extracting generalized knowledge (semantic memory). Previous research comparing a unitary vs. a bi-partite account of memory in an SEM framework has supported the idea of separable memory factors underlying declarative memory, at least in adults (Nyberg, 1994).

Adopting a process-oriented view, recent computational models of memory have argued for a functionalist distinction between memory specificity and generalization through a labor division between the hippocampus and the cortex (McClelland et al., 1995; Norman & O’Reilly, 2003). Here, a set of neurocomputations support different memory demands. Important to learning specific episodes are pattern separation that discriminates between similar memories through the reduction of representational similarity, and pattern completion that retrieves linked associations among co-occurring elements (Marr, 1971; Norman & O’Reilly, 2003; Rolls, 2016). Both of these computations are thought to be specialties of the hippocampus. In contrast, the cortex is well suited to slowly learn statistical regularities that enables generalization (McClelland et al., 1995; Norman & O’Reilly, 2003). Interestingly, rapid generalization also relies on the hippocampus, either via retrieval mechanisms of related episodes (Kumaran & McClelland, 2012) or via a distributed coding scheme carried by a specific subset of the hippocampal circuitry (Schapiro et al., 2017). From this vantage point, memory abilities take shape of a tri-partite structure, encompassing pattern separation, pattern completion, and generalization as three separable mnemonic processes.

**Fig. 1:**
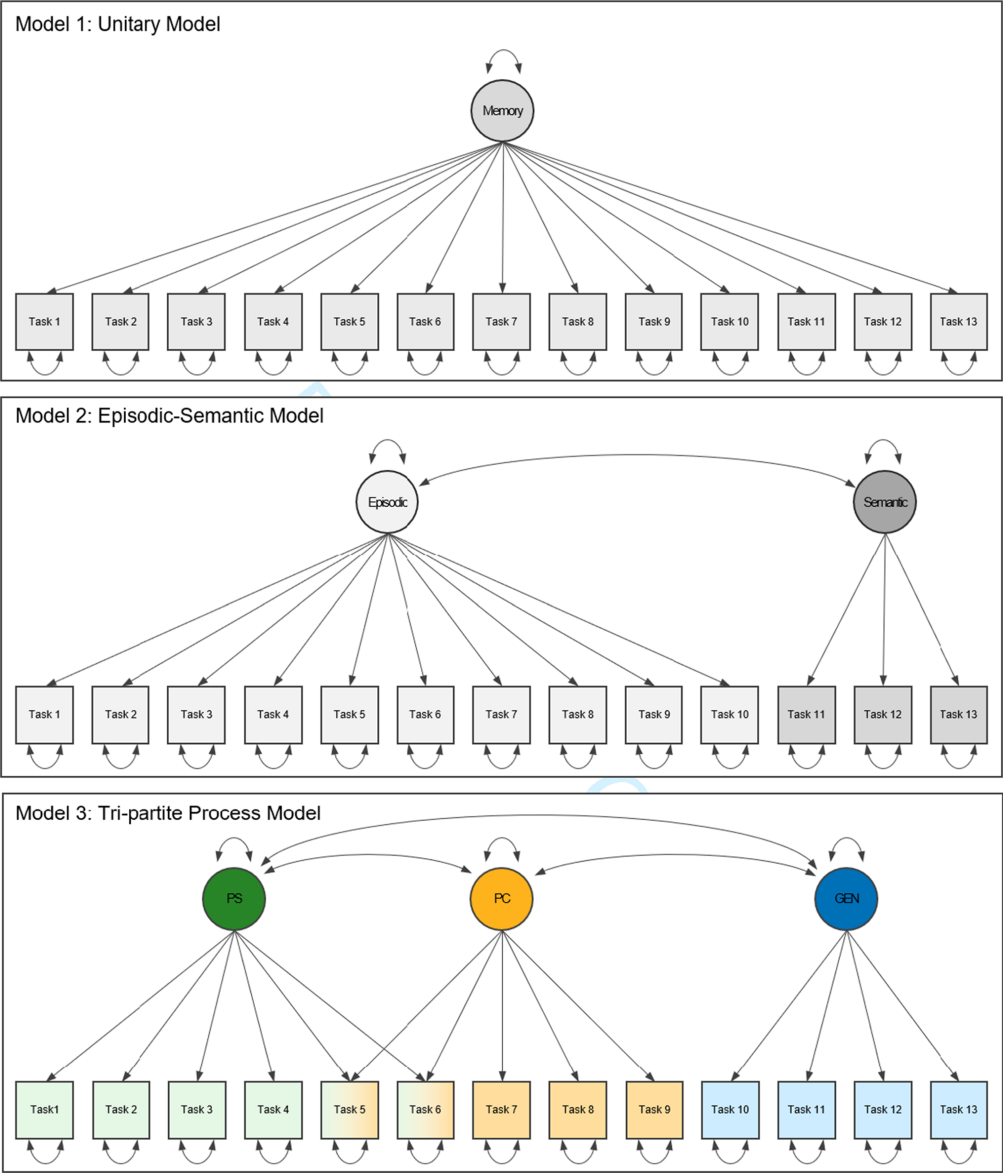
Path diagrams of three competing SEMs on the structure of memory. *Squares* represent observed performance measures on the behavioral tasks. *Circles* represent latent constructs (PS = Pattern Separation, PC = Pattern Completion, GEN = Generalization). *Single-headed arrows* indicate regressions and *double-headed arrows* indicate covariances. See Buchberger et al., (in press) for an in-depth description of the models

Which of these views best explains memory abilities across early development? This question requires adequate statistical methods that can adjudicate among multiple competing ideas on the underlying structure of memory. The simulation in the following section of this paper will address methodological challenges in study planning when aiming to compare three hypothetical models: a unitary model (Model 1), a bi-partite model (Model 2), and a tri-partite model (Model 3) on the compositional structure of memory (Fig. 1). Specifically, we will focus on the issue of estimating statistical power for this model comparison. Here, we address the impact of two main aspects in particular: (i) the separability of the theoretical constructs, i.e., inter-relatedness of the latent factors in the competing models and (ii) the reliability of the measures, i.e., the loadings of the manifest variables. Details on the theoretical basis and the selection of methodological indicators for each model can be found in Buchberger et al. (in press). Important for this context is the fact that our specification of Models 2 and 3 results in a non-nested model comparison (due to the cross-loadings for the latent factors pattern separation and pattern completion and different allocation of the indicator Task 10). Therefore a priori estimations of statistical power for selecting the correct model call for a simulation-based approach.

## Methods

In the following, we demonstrate how to conduct a simulation-based power analysis for non-nested model comparisons. Here, we refer to statistical power as the probability of correctly selecting a model given a selection of candidate models. In a hypothesis testing framework, the alternative hypothesis corresponds to a given model being the true model. Statistical power then reflects the correct selection rate of a model and the type I error corresponds to the probability of incorrectly selecting that model when one of the other candidates is the true model. For the sake of illustration, we will refer to the example on the compositional structure of memory outlined in “Example: Competing modelson the compositional structure of memory” throughout. For this example, we aim to derive estimates of required sample size to identify the tri-partite model as the best-fitting model among the three competing models, if it indeed generated the data. In a step-by-step guide, we therefore show how to specify the competing models in R, how to set up different design conditions for the simulation, how to define the functions to generate, analyze, and summarize synthetic data and how to execute the simulation. Based on two separate simulations, we show how the separability of theoretical constructs (“What is the impact of the separability of theoretical mod-els on estimates of statistical power?”) and the reliability of the manifest variables (“What is the impact of the reliabil-ity of manifest variables on estimates of statistical power?”) impacts estimates of statistical power for model comparisons by varying inter-factor correlations and factor loadings across different conditions of the simulation. Finally, we employ the same simulation-based approach to investigate type I error rates, that is erroneously identifying the tri-partite model as the best-fitting model if the bi-partite or unitary model generated the data (“How can simulations inform us about typeI error rates?”).

### How does a simulation work: Power-estimations for the comparison of non-nested SEMs

**Fig. 2:**
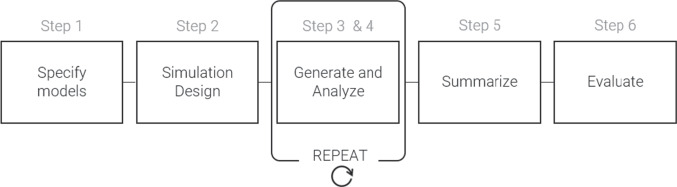
Schematic overview of the simulation process

To investigate statistical power for deciding between competing theoretical non-nested models, we suggest a Monte Carlo simulation-based approach (Metropolis, Rosenbluth, Rosenbluth, Teller, & Teller, 1953). Monte Carlo studies represent a computer-intensive simulation, which allows to approximate statistical power for a given study design (Muthén & Muthén, 2002). While Monte Carlo simulations can be implemented for a variety of research questions, the flexibility of the approach makes them especially well suited for obtaining a priori power estimates for the comparison of non-nested SEMs. Furthermore, analytical approaches are tied to assumptions (e.g., no missing data, multivariate normality), whereas simulation-based approaches allow for arbitrary data generating processes. In Monte Carlo studies, synthetic data are repeatedly generated with a set of different hypothesized parameter values and analyzed across all samples. Summary statistics from the entirety of the simulated datasets are then used to draw conclusions (Muthén & Muthén, 2002; Paxton, Curran, Bollen, Kirby, & Chen, 2001). The general procedure of Monte Carlo simulations follows a common core structure: (i) generate – in which multiple synthetic datasets are generated based on the hypothesized models, (ii) analyze – in which the synthetic data are analyzed for every iteration of the simulation, (iii) summarize – in which the results are pooled over all simulation iterations. This general procedure is applicable to a variety of scenarios. When comparing competing SEMs, it is necessary to specify the competing theoretical models, and decide on the simulation design, that is which model parameters of interest should be modified across the simulations. Finally, researchers need to evaluate the results from the simulation in order to derive practical implications from the simulation (see Fig. 2). While these steps can be manually coded in a statistical research software, such as R, the implementation of a simulation can become increasingly complex and error-prone with increasing number of conditions that are being simulated. A useful tool that can guide novice simulators in setting up a Monte Carlo simulation is the R package simDesign (Chalmers & Adkins, 2020), which facilitates the implementation of the internal logic of generate – analyze – simulate and can accommodate various research designs. All simulation steps in this study were therefore implemented within the simDesign package.

#### Step 1: Specifying competing SEMs

The first step of the simulation is translating the theoretical assumptions about latent constructs of interest and the manifest variables into an SEM. This step entails (a) specifying the number of latent factors (how many theoretical constructs are thought to influence the observed behavior in the manifest variables?), and (b) the allocation of each manifest variable to (at least one) latent variable (which of the manifest variables captures the respective latent construct?). In R, the structure of a SEM can be easily implemented using lavaan syntax (Rosseel, 2012). Typically, useful operators for specifying a model entail factor loadings (=~), (co-)variances (~~), and means or intercepts (~1). The tri-partite model (Fig. 1, Model 3) could be specified in lavaan syntax as the following:
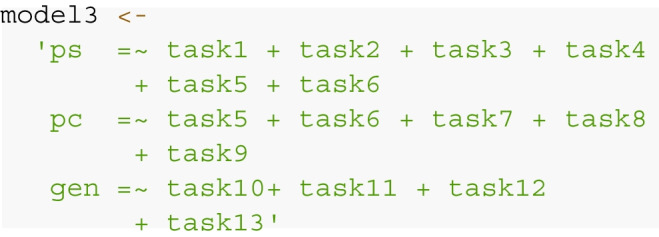


The model is defined by a model string, which specifies three latent factors of memory (Pattern Separation [ps]; Pattern Completion, [pc]; Generalization, [gen]) which are measured through the manifest variables (tasks 1 to 13). Note that lavaan entails several user-friendly functions for fitting models that automatically include default settings for model components that are not explicitly specified (e.g., adding residual variances, covariances of exogenous latent variables). Thus, the model syntax can be kept very concise (check documentation of the respective function for full information on default settings).

Estimating statistical power for model comparisons requires that researchers specify each of the competing models and all aspects in which they differ from one another (e.g., in number of latent factors, allocations of manifest variables; For specifications of Models 1 and 2, see supplementary R code on GitHub, Buchberger, Ngo, Peikert, Brandmaier, & Werkle-Bergner, 2024).

#### Step 2: Simulation design

When defining the structure of the theoretical model for data generation, it is crucial to specify all parameters in the model, particularly the loading strength of the manifest variables, along with the means and (co-)variances of the latent factors. This step also requires the specification of which parameter values should be kept constant or which should be modified across different simulation iterations. Parameters for which there are strong assumptions (e.g., derived from previous work) can be entered in the models and kept constant. Parameters for which the impact on statistical power is of interest should be included as a condition in the simulation design. For example, if the question pertains to how the reliability of the measures may impact the estimates of required sample size, different levels of loading strength can be included in the conditions for which data is being simulated. The createDesign() function of the simDesign package can be used to create a full list of conditions for the simulation. All parameters that should be varied in the simulation are included as arguments in the createDesign() function. Full crossings of all possible values for the different parameters are then generated as conditions for the simulation. Note, however, that the number of simulation conditions and therefore the required computation time increases exponentially for full combinations of all simulation conditions. Researchers should thus be mindful about the number of conditions in a given simulation to avoid overly computationally intensive simulations, which can further be increasingly difficult to interpret. If only specific combinations of the parameters in the simulation are of interest for a given research question, the resulting design data frame can be adjusted accordingly, e.g., by deleting specific rows from the data frame. Below is our design data frame that includes different simulation conditions for (i) the loading strength of the manifest variables and (ii) the sample sizes:
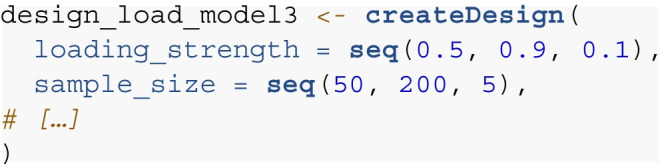
 Here, the design for the Monte Carlo simulations consists of five loading strength conditions (i.e., 0.5 to 0.9 in increments of 0.1). The sample size is varied from 50 to 200 in increments of 5. In total, data for 155 conditions (5 × 31) are generated.

#### Step 3: Generate

Next, we define the mechanism to generate the synthetic data. An easy way to generate synthetic data within an SEM framework in R is the simulateData() function available in the lavaan package (Rosseel, 2012). In the example on memory composition, we simulate multivariate normal data, as the indicators represent continuous values of task performance from the memory tasks. However, the simulation approach presented here can of course be extended to dichotomous or polytomous data for other use cases. Within the generate function, all parameters that are predetermined across iterations can be directly entered into the generating model syntax. However, all parameters that are supposed to be varied between different iterations of the simulation need to be accessed through the condition argument that is returned through the createDesign() function in step 2. It can be useful to examine the resulting datasets prior to running the simulation to assess whether the data generating mechanism returns realistic datasets (e.g., regarding the variability of the data, extreme values), to allow for refining the generate function accordingly (similar to prior predictive checks as often suggested in the context of Bayesian approaches, Gelman et al., 2020). Given our interests in the effect of loading strength and strength of covariance between the latent factors, we define these parameters in step 2 (Design) such that they can now be called via the condition argument in the generate function:
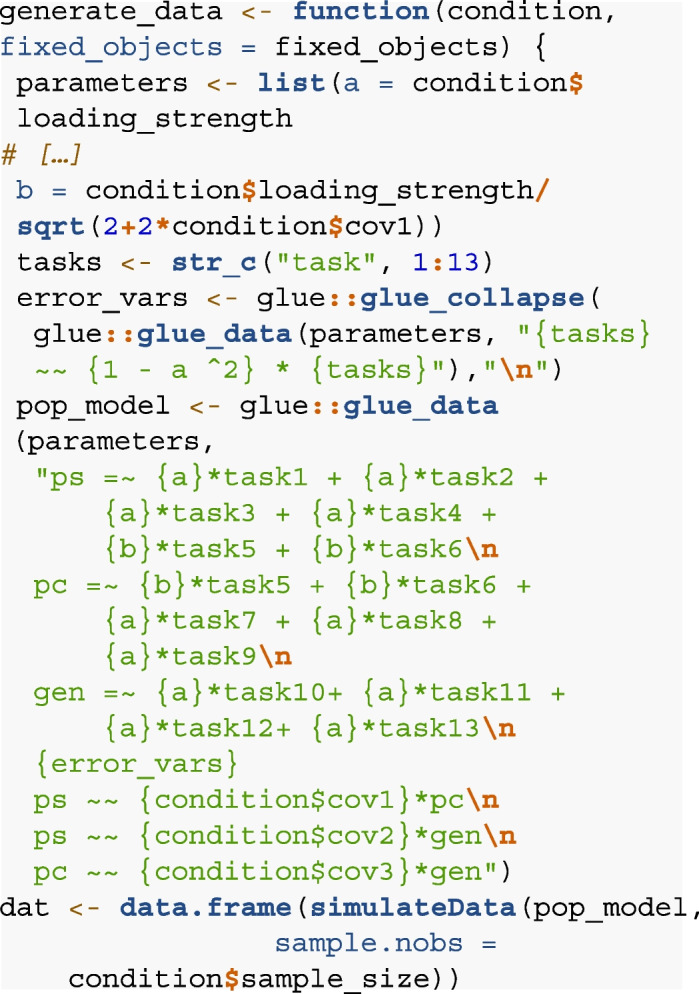


Here, we define the generate function for the tripartite model. The factor loadings specified in step 2 (Design) are called from the condition argument such that they are varied across different iterations of the simulation, and are defined as “a” for syntax readability. For standardization purposes (that is, the total variance of each manifest variable summing up to 1), the loadings of indicators Task5 and Task6 are set to $\frac{a}{\sqrt{(2+2 \cdot cov1)}}$ to account for cross-loadings on multiple factors and are defined as “b” to ease syntax readability (cov1 = covariance between the latent factors pattern separation and pattern completion). Both loading parameters a and b are stored in the list “parameters” such that they can be called via the glue_data function from the glue package (Hester & Bryan, 2022) to concatenate the model string. The residual variances of the manifest variables are set to $1-a^2$ for standardization purposes. The population model is defined by a model string, which specifies three latent factors (ps, pc, gen) measured by the 13 manifest indicators (ps: tasks 1 to 6, pc: tasks 5 to 9, gen: tasks 10 to 13), the residual variances as defined above and the covariance between the latent factors as defined in the condition argument. The data are simulated from the model syntax via the simulateData() function from the lavaan package (Rosseel, 2012) and stored as “dat”.

Note that the Monte Carlo simulation method does not limit researchers to the simulation of normally distributed data, but can be used with any form of data-generating mechanism. Further, the simulation-based approach allows researchers to anticipate and investigate the effects of (planned) missingness in the data on estimates for statistical power (Brandmaier, 2020; Schoemann, Miller, Pornprasertmanit, & Wu, 2014). Here, different amounts and patterns of missingness can be entered into the simulation by removing data from the generated datasets. The resulting data can then be analyzed and summarized to investigate the effects of attrition, planned or random missingness on the estimates for statistical power. The implementation of these characteristics of the data is beyond the scope of this tutorial. However, in research scenarios in which violations of the normality assumption or missing data can be expected, researchers should consider a simulation-based approach to estimate statistical power, as the approach presented here is free from any assumptions on the data distribution.

#### Step 4: Analyze

In this step, we define the analyze function, which specifies how the data generated in step 3 should be analyzed. For the comparison of non-nested models, we rely on a combination of relative and absolute model fit parameters (for discussion of an alternative model selection approach, see “Discussionand conclusion”). We fit each of the competing models to each of the simulated data sets using the sem() function from the lavaan package (Rosseel, 2012) with a maximum likelihood estimator and extract different measures of model fit using the helper function get_fitmeasures() (see funs.R in the supplementary material on GitHub, Buchberger et al., 2024), namely the Comparative Fit Index (CFI, Bentler, 1990), root mean square error of approximation (RMSEA, Steiger, 2016) and the Bayesian information criterion (BIC, Raftery, 1986). The function returns the fit measures, the simulation model that was used in the respective iteration and information on model convergence. The analyze function for the example on memory composition could thus look like this:
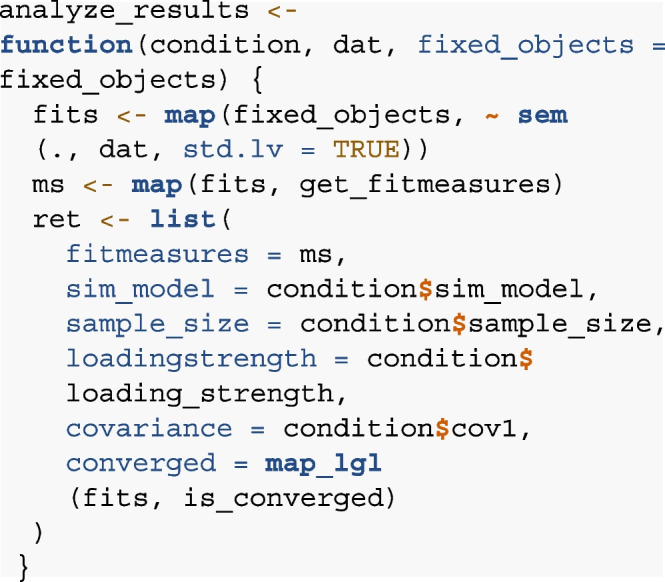


#### Step 5: Summarize

In step 5, we define how the results from the analyze step should be summarized. To this end, we extract how often the model used to generate the data (step 2) was actually recovered as the best-fitting model. We implemented the following decision rules for identifying the best-fitting model: (i) We define cut-off criteria for an adequate model fit according to established rules as CFI > .95 and RMSEA < 0.06 (Hu & Bentler, 1999). (ii) Among the models that meet the cut-off criteria, we select the best- fitting model for each data set based on the relative model fit (lowest BIC value). The goal of (i) and (ii) is to check whether the ground truth model that generated the data can be identified as the best-fitting model in a given simulation run. For cases in which a given dataset does not converge for one of the models, we consider the outcome of these iterations as failing to recover the correct model. We summarize the proportion of successful model recovery for each combination of model parameters in the simulation separately. The summarize function that concatenates the results across simulation iterations for the example on the structure of memory in childhood could thus look like this:
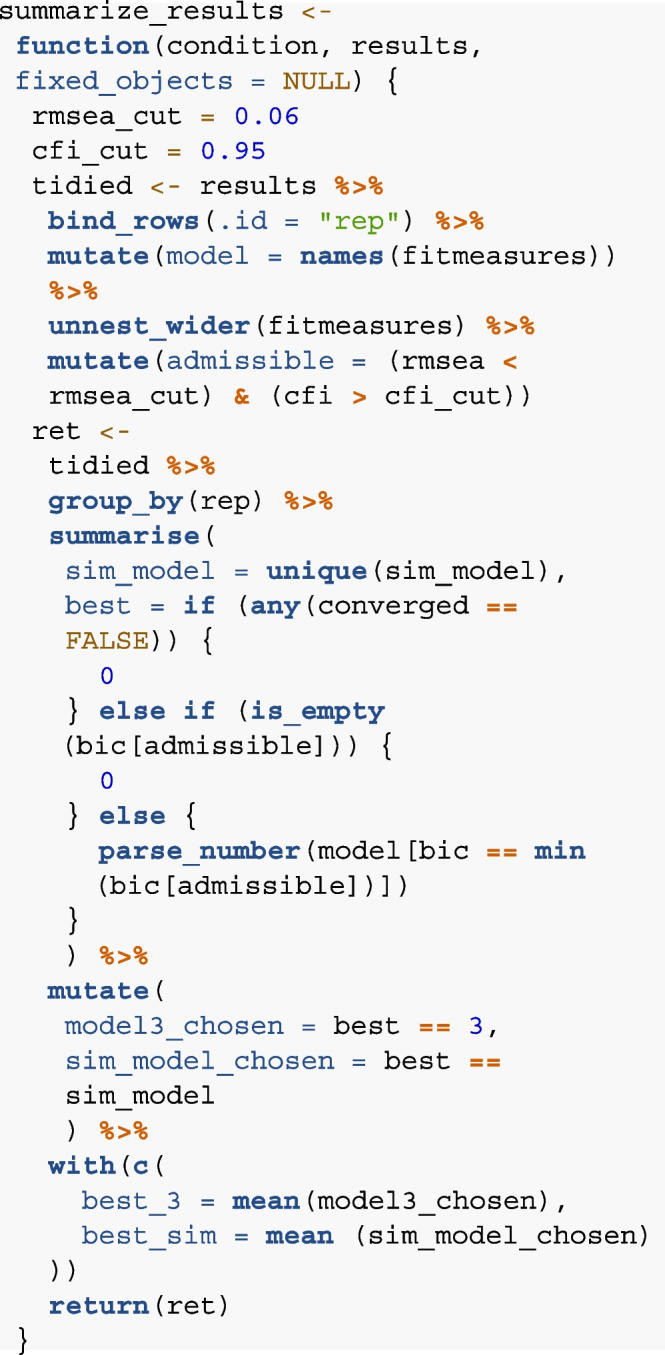


First, the function restructures the results from the analyze function to obtain a tidy data frame with the fit measures for each model in a separate row. This data is then summarized for each iteration. The function identifies (a) the ground truth model that generated the data in this simulation iteration (sim_model) and (b) the model which produced the best model fit (best). Here, the best model is defined as “0” if one of the three theoretical models did not converge, or if none of the models met the cut-off criteria as defined above. Otherwise, the best model is selected among the ones that met the cut-off criteria based on the relative model fit. The function returns the argument best_3, indicating how often across all iterations Model 3 was identified as the best-fitting model, and best_sim, indicating how often the model that simulated the data was identified as the best-fitting model (note that in the current analysis, both values are identical. best_sim can however be useful to investigate type I error, see “How cansimulations inform us about type I error rates?”).

#### Step 6: Running and evaluating the simulation

In a final step, we call the runSimulation() function from the simDesign package to execute the consecutive steps outlined above. Running the simulation for our example could look like this:
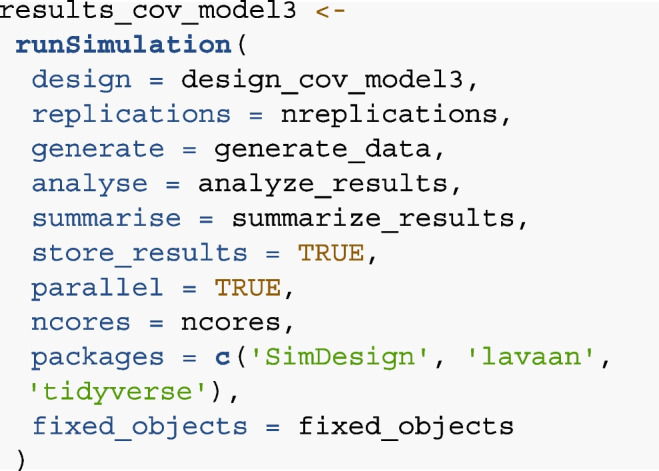


In this function, the pre-defined functions generate_data(), analyze_results() and summarise_results() are called. The number of replications is specified in the replications argument. To ensure reasonable stability of the results, a sufficient number of replications is recommended. In our example, we simulated 1000 datasets per condition. This leads to a standard error of the simulated power of less than 0.5%. The store_results argument can be used to store the individual results generated by the summarize function, allowing users to investigate the outcome on every iteration (in this example, the data frame containing all fit measures and convergence information for each iteration). Note, however, that storing the individual results increases computation time and size of the resulting outcome arguments. The argument parallel can be used for parallel processing across multiple cores (e.g., when running the analysis on a computer cluster) to reduce computation time. If not specified otherwise, all available cores will be used per default when parallel = TRUE.

**Fig. 3:**
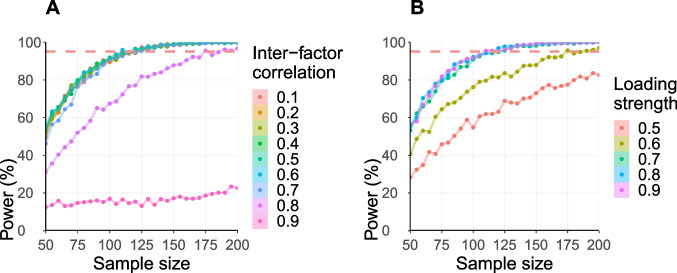
Simulation results: Estimated statistical power for correctly selecting the data-generating model, i.e., Model 3 (*y*-axis) as a function of sample size (*x*-axis) with varying the factor inter-correlations when the loading strength was fixed to 0.7 (**A**) ;  and with varying the loading strength when the factor inter-correlation was fixed to 0.3 (**B**). For each combination of parameters, 1000 datasets were simulated. The *red dashed horizontal line* positioned at 95% discovery rate reflects the cut-off for identifying the appropriate sample size. The syntax to generate these plots with ggplot() can be found in the supplementary R Code on GitHub

The output can then be used to examine power curves (see Fig. 3) for each of the parameters of interest in the simulation. Power curves are line plots that depict how changing one variable (e.g., sample size) would affect the power of the test. In the example, we can plot power curves demonstrating the impact of the separability of the theoretical constructs, implemented as the strength of the covariance between the latent factors, or the impact of the reliability of the measures, implemented as the strength of the loadings, on power estimation. Importantly, these data reveal the relative importance of different model parameters on sample size requirements. To obtain easily readable outputs, we plot the sample size from the simulation (*x*-axis) against the frequency with which the data-generating model was correctly recovered as the best-fitting model (*y*-axis). To derive estimates on the required sample size in the example presented here, we aimed for a power of 95% in recovering the ground truth model as the best-fitting model.

## Results: Statistical power estimates for investigating the compositional structure of memory

### What is the impact of the separability of theoretical models on estimates of statistical power?

In the example of comparing competing models of memory structure, we first examined the separability of the theoretical models by investigating the influence of the latent factors’ correlations on statistical power estimates. In our example simulation, we investigated the statistical power to retrieve Model 3 as the best-fitting model, when it indeed generated the data, while varying sample sizes from 50 to 200 and factor inter-correlations from 0.1 to 0.9. In this case, we restricted the simulated correlations between the latent factors to positive values, as there is no indication in the literature to suspect a negative relation among these memory capacities (e.g., Tucker-Drob, Brandmaier, & Lindenberger, 2019). Figure 3A displays the obtained power curves illustrating the estimated statistical power dependent on the total sample size and the factor inter-correlation. For this estimate, the loading of the manifest variables was fixed at 0.7 and the inter-correlation between all latent factors were varied simultaneously.

As expected, statistical power increased with increasing sample size. However, the separability of the latent factors mattered: The higher the correlations between factors, the less able we were to distinguish multi-factor models from a one-factor model. That is, the smaller the difference between competing models, the greater the sample size that would be required to dissociate between them with adequate power. For very high levels of inter-correlation (0.8 and higher) the increase of statistical power with increasing sample size was remarkably lower than for lower levels of inter-correlation (0.7 and below). When the inter-factor correlation was 0.9, not even a larger sample size (*N* =200) achieved an adequate level of power of 80%. These results are not surprising given that increasing the inter-correlations between the latent factors renders the models more similar to each other or – in the case of correlations approaching 1 – identical, making their distinction impossible. For moderate levels of inter-correlation ($<= 0.7$) a statistical power of 95% was reached for *N* = 125.

### What is the impact of the reliability of manifest variables on estimates of statistical power?

In a second step, we further examined the effect of the construct reliability on statistical power by investigating the loading strength of the manifest variables. To this end, we conducted a second simulation following the steps outlined above while varying the sample size from 50 to 200 and the loadings of the manifest variables from 0.5 to 0.9. Figure 3B displays the obtained power curves, illustrating statistical power dependent on total sample size and loading strength of the manifest variables. For this estimate, the factor inter-correlation was set to 0.3. Again, increasing sample size led to higher statistical power. However, the loading strength impacted estimates of statistical power: the higher the loadings of the manifest variables, the higher the statistical power achieved by a given sample size. For reasonably high levels of factor loadings (>= 0.7), a statistical power of 95% was reached for *N* = 125.

### How can simulations inform us about type I error rates?

**Fig. 4:**
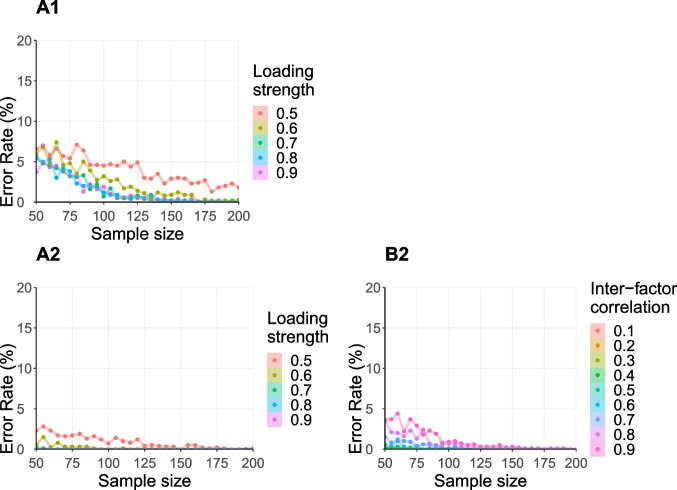
Simulation results: Probability to erroneously pick Model 3 as the best-fitting model when indeed Model 1 (A1) or Model 2 (A2/B2) generated the data, as a function of total sample size (*x*-axis) and the loadings strength of the manifest variables (A1/A2) or the strength of the covariance of the latent factors (B2). Note that the *y*-axis reaches from 0 to 20% for the sake of visibility

The main part of this tutorial has focused on estimating statistical power that is required to reject a null hypothesis when it is indeed false. However, it is necessary to also consider the reverse – rejecting a null hypothesis that is in fact true (type I error). In our example, type I error would be committed by misidentifying Model 3 as the best-fitting model when in reality, the data were generated from either Models 1 or 2. We can leverage the simulation approach to quantify type I error rates dependent on the different simulation conditions (e.g., inter-factor correlations, factor loadings). We conducted an analogous analysis to the one outlined above (“Step 1: Specifying competing SEMs” – “Step 6: Running and evaluat-ing the simulation”), with the difference that Models 1 and 2 are defined as the generating population models (step 3, “Step 3: Generate”). The outcomes of this analysis show the probability of erroneously identifying an overly complex model, when the more simplistic models generated the data (see Fig. 4).

The results of this analysis reveal that even for low levels of loadings strength ($< 0.7$), the probability of erroneously picking Model 3 as the best-fitting model is reasonably low (< 8%). Likewise, this probability remains reasonably low for different levels of inter-factor correlations for all sample sizes (< 5%). Thus, in this specific model comparison type I error probability does not appear to be the main aspect driving sample size requirements for an informative research design.

### Deriving a sample estimate from the simulation results

To derive a joint sample size estimate from the different parameters in the simulation, it is crucial to reiterate the theoretical assumptions on the strength of all parameters investigated in the simulation. In our illustration, it is reasonable to assume that the latent factors in the tri-partite model (Model 3, Fig. 1) are only moderately correlated (below 0.7). Therefore, our model comparison requires a sample size of *N* = 125 to reach statistical power of 95% to adjudicate between the theoretical models, if the hypothesized model (Model 3) generates the data. We further assume that the manifest variables capture the latent constructs reasonably well – that is, exerting a loading strength of 0.7 or above. The results of the simulation suggest that, again, *N* = 125 would suffice to achieve 95% with regard to the reliability of the manifest factors. In our example, the results of both analyses align, such that a sample size of *N* = 125 suffices to achieve a power of 95% regarding both the separability of the theoretical constructs and the reliability of the indicators. In cases where the derived sample estimate differs between the two analyses we suggest deriving the more conservative estimate of required sample size from the two simulations. The results from the type I error analysis further indicate that the probability of erroneously choosing an overly complex model is generally low. Therefore, the probability of committing a type I error for the sample size derived from the simulations on model separability and measurement reliability (*N* = 125) is negligible.

## Discussion and conclusion

SEM offers a powerful tool for modeling multivariate relationships between psychological constructs. Maximizing its utility in psychological sciences hinges on the integration of its complex characteristics with power estimation – another key tenet of scientific rigor. Our work exemplifies a roadmap for navigating some of the challenges in harmonizing these two efforts by implementing a priori power estimation within an SEM framework. In the case of information-theoretic model comparisons for non-nested models, statistical power cannot be determined based on traditional $\chi ^2$-based statistics. Monte Carlo simulations provide a useful tool to circumvent this methodological challenge (Muthén & Muthén, 2002), allowing researchers to derive informed decisions on sample size planning to ensure sufficient statistical power and prevent uninformative or misleading research (Button et al., 2013). In this study, we illustrate a step-by-step approach to implementing a Monte Carlo simulation in the statistical programming language R to estimate the required sample size for model comparisons of non-nested models. The complete code is available on GitHub (Buchberger et al., 2024) to yield a starting point for researchers who want to implement their own Monte Carlo power simulations.

The results from our illustration highlight the importance of two model parameters: the separability of the theoretical models on the basis of factor inter-correlation, and the reliability with which the construct can be measured on the basis of the loading strength of the manifest variables. These results align with theoretical knowledge on SEM (Wolf et al., 2013), but they provide additional insights on the tangible impact of these parameters on statistical power estimation in the specific research design discussed here and in other SEMs more broadly.

First, we showed that detecting differences in model fit requires increasingly many participants the higher the inter-correlation of the factors turns out practically. Therefore, it is warranted to consider the actual separability of the constructs in question to avoid underpowered sampling plans. We acknowledge that committing to specific parameter values, such as factor inter-correlations, can be difficult when setting up the population models for the simulations. Thus, we recommend researchers to consult existing literature in order to make informed assumptions on the relationship among different latent factors. For cases in which no or little evidence exists on the relationship between the constructs under investigation, we recommend to enter these inter-correlations into the simulation as parameter of interest (as demonstrated in the example of this paper) and to then carefully evaluate what level of resolution is required when separating inter-related factors depending on practical relevance.

Second, the results on the loadings of manifest variables stresses the importance of employing indicators that capture the latent construct with high reliability (Zuo, Xu, & Milham, 2019). Considering the issue of reliability is a crucial step in experimental design, as the reliability of the indicators heavily influences the practical ability to identify the targeted latent construct with sufficient precision and differentiate between competing models. Again, committing to population values can be difficult, especially if there is no prior work from which to draw values. In this case, we still advise to estimate plausible values from related scientific areas. For instance, selecting factor loading strength in the tri-partite model is not straightforward. However, we can do a literature search on similar models and take the reported test reliability (e.g., a test–retest) or construct reliability (e.g., coefficient alpha) and translate this back into factor loadings strength and residual loadings (see Brandmaier et al., 2018 for examples on how to parametrize longitudinal models from minimal available information). If information about test reliability is available, we set the loadings to the square-root of the reliability to take this prior information into account. Then, this yields the following construct reliability for our design (assuming identical loadings across *k* items):

1 $$\begin{aligned} \alpha = \frac{\sum _{k}{ \lambda ^2}}{\sum _{k}{\lambda ^2 + 1 - \lambda ^2 }} \end{aligned}$$

If instead, prior knowledge about construct reliability exists and this reliability estimate $\alpha$ was based on *k* items, we solve Eq. 1 for $\lambda$ and obtain an estimate that may serve to inform our factor loadings:

2 $$\begin{aligned} \lambda = \sqrt{\frac{\alpha }{\alpha + k - \alpha \cdot k }} \end{aligned}$$

For example, taking from a literature search that the construct reliability of the construct of interest (reported as Cronbach’s alpha) was 0.7 and the estimate was based on *k*=4 tests, we set our loadings to $\lambda$ = 0.61 (using Eq. 2). If a study reported a test–retest correlation of a single test to be *r*=0.8, then we set the factor loading to $\lambda = \sqrt{0.8} = 0.64$. If in doubt, we suggest using conservative values rather than too liberal values to avoid underpowered studies.

Note that in this tutorial, we limited our analyses to investigating the impact of either the separability of the theoretical constructs or the impact of the reliability of the estimates by keeping the other parameter constant throughout the simulation. The interaction of these parameters may differentially impact the resultant sample size estimates. However, a thorough investigation of the interaction of the two influencing factors would exponentially increase the number of conditions in a simulation. In our example on memory component processes, we therefore confined our simulation to separate analyses in the interest of conciseness. For interested readers, two additional analyses on (a) the effect of loading strength on estimates of power for higher levels of inter-factor correlation (0.7 as opposed to 0.3 in the original simulation) and (b) the effect of inter-factor correlation on estimates of power for lower levels of factor-loadings (0.5 as opposed to 0.7 in the original simulation) can be found in the appendix of this manuscript. Given that previous work on memory development supports the assumption of reasonably high factor loadings and moderate inter-factor correlation in the present example, these additional analyses leave our conclusions unchanged.

Further, we have presented an approach that leverages a combination of absolute and relative model fit parameters to adjudicate between competing models. While such parameter-based decisions are well established in the field, they may result in choosing one model over the other, even in cases when differences in model fit parameters are very small, as one can never conclude that two models fit equally well or that there is too little data to make a decision with enough confidence. An alternative approach to this criterion for model selection originates from a theory by Vuong (1989), who suggested likelihood-ratio tests for non-nested models. Recent advances that build on this idea have postulated a framework that allows to test hypotheses of model distinguishability and difference in fit (Levy & Hancock, 2007, 2011), which can be applied to non-nested SEMs and easily implemented via new software packages in R (nonnest2, Merkle, You, & Preacher, 2016). The resulting recommendations for the comparison of non-nested SEMs posit a stepwise procedure to test whether competing non-nested models are distinguishable in a given population and, if yes, whether one fits the data significantly better than the other. Importantly, this method derives interval estimates for differences in non-nested information criteria (Merkle et al., 2016). Thus, this approach not only allows concluding that one or the other model fits the data better but also allows for the possibility to conclude that there is insufficient evidence to determine which of two models fits a given dataset better. This stepwise procedure can be implemented as a model selection criterion in a power simulation like the one presented above. To integrate model selection via a likelihood-ratio test for non-nested models into the current simulation, one would need to adapt the summarize function accordingly, such that it identifies model 3 as the best-fitting model only if (a) the competing models are distinguishable on the simulated data set of a given iteration, (b) the likelihood-ratio test for non-nested models indicates a significantly better fit for Model 3 compared to Models 1 and 2 and (c) Model 3 reaches the cutoffs for absolute model fit, as defined in the previous simulation. The vuongtest() function from the nonnest2 package (Merkle & You, 2014) can be a helpful tool for integrating these tests into the simulation. While a systematic investigation of the interplay of indicator reliability, separability of latent factors and statistical power for model selection via model fit parameters versus the likelihood-ratio test for non-nested models is beyond the scope of this tutorial, it is worth noting that such an alternative approach may yield better model recovery in certain cases. The conditions under which model selection via the likelihood-ratio test for non-nested models provides better estimates of statistical power are thus an interesting avenue for future research.

While we demonstrate in this tutorial that simulation-based techniques help overcome methodological shortcomings from traditional analytical approaches, the randomness that is inherent to such approaches may seem at odds with efforts in results reproducibility and replicability. Here, a clear-cut solution is through transparent and accessible documentation of the simulation code and adequate software management. That is, researchers should make use of repositories on platforms such as GitHub or the Open Science Framework to make code scripts available to other researchers, and clearly specify the versions of software programs and packages that were used (e.g., via containerization or at least in written form). We thus urge researchers to seriously consider the issue of reproducibility for their simulation analyses to make outcomes of such a priori power estimates easily accessible to other researchers and reviewers (for a more detailed guideline on a reproducible workflow in R see Peikert, Lissa, & Brandmaier, 2021 and the implementation of this manuscript on GitHub as an example). Note, however, that – specifically in cases when simulations are parallelized on computing clusters – the exact reproducibility of simulations as demonstrated in this tutorial might be compromised, even when a seed is specified in the analysis script. Nevertheless, a sufficiently high number of iterations ensures replicability, i.e., comparable estimates of statistical power. Derived conclusions about required sample size will therefore remain unaffected.

In sum, we argue that sample size planning in the case of non-nested SEM comparisons can be achieved via a simulation-based approach. In particular, we highlighted that the separability of theoretical constructs, as well as the reliability of the measures, have a major impact on estimates of statistical power. To this end, we hope that this tutorial advances the use of simulation-based approaches to estimating statistical power for model selection when comparing non-nested models in an SEM framework.

## Open Practices Statement

The supplementary materials for this article (including the data and R scripts) are publicly available on GitHub (Buchberger et al., 2024). The analyses reported in this manuscript were not preregistered.

## Figures and Tables

**Fig. 5:**
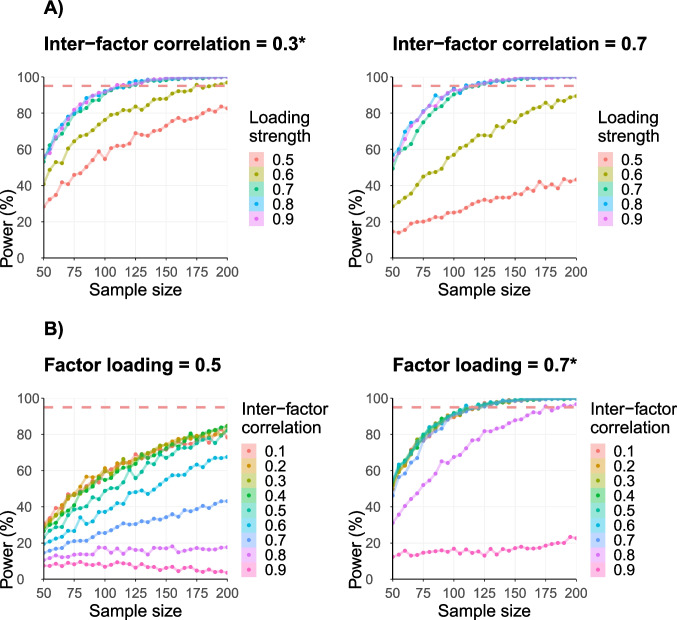
Estimated statistical power for correctly selecting the data-generating model (*y*-axis) as a function of sample size (*x*-axis) depending on (**A**) loading strength for lower versus higher inter-factor correlation and (**B**) inter-factor correlation for lower versus higher factor loadings. * denotes results from the original analyses in the main manuscript
